# [^11^C]MK-6884 PET imaging reveals lower M_4_ muscarinic acetylcholine receptor availability following cocaine self-administration in male rats

**DOI:** 10.1007/s43440-025-00695-9

**Published:** 2025-01-14

**Authors:** Krishna K. Gollapelli, Ivan Krizan, Bhuvanachandra Bhoopal, Naresh Damuka, Carson Moriarty, Mack Miller, Kiran K. Solingapuram Sai, Robert W. Gould

**Affiliations:** 1 Department of Radiology, Wake Forest University School of Medicine, Winston-Salem, NC USA; 2 Department of Translational Neuroscience, Center for Addiction Research, Wake Forest University School of Medicine, 115 South Chestnut St, Winston-Salem, NC 27101 USA

**Keywords:** Cocaine use disorder (CUD), Cocaine self-administration, PET imaging, [^11^C]MK-6884, Radiochemistry, M_4_ mAChR

## Abstract

**Background:**

Cocaine Use Disorder (CUD) remains a significant problem in the United States, with high rates of relapse and no present FDA-approved treatment. The acetylcholine neurotransmitter system, specifically through modulation of muscarinic acetylcholine receptor (mAChR) function, has shown promise as a therapeutic target for multiple aspects of CUD. Enhancement of the M_4_ mAChR subtype via positive allosteric modulation has been shown to inhibit the behavioral and neurochemical effects of cocaine across several rodent models of CUD. However, it is unclear how cocaine exposure affects M_4_ mAChR expression or distribution.

**Objectives:**

To evaluate the effects of cocaine self-administration on M_4_ mAChR availability using [^11^C]MK-6884 in vivo PET imaging in rats that self-administered cocaine (cocaine SA) or sucrose pellets (control).

**Methods:**

Sprague-Dawley rats self-administered cocaine or sucrose pellets for 15 days under 2-h or 4-h sessions followed by PET imaging with [^11^C]MK-6884, a radiolabeled M_4_ selective positive allosteric modulator to determine the effects of cocaine on [^11^C]MK-6884 standard uptake values with cerebellum as reference (SUVr).

**Results:**

Cumulative cocaine intake ranged between 324 and 776 mg/kg. Cocaine self-administration was associated with significantly lower [^11^C]MK-6884 SUVrs in the cortex, hippocampus, and striatum compared to cocaine-naive rats, with a negative correlation between radiotracer SUVrs and cocaine intake in the hippocampus.

**Conclusions:**

These results suggest that cocaine self-administration decreases M_4_ mAChR availability, providing further support for pursuing activation/enhancement of M_4_ mAChR function as a viable pharmacotherapeutic approach for CUD.

**Supplementary Information:**

The online version contains supplementary material available at 10.1007/s43440-025-00695-9.

## Introduction

Cocaine Use Disorder (CUD) is a serious public health issue with no effective pharmacological treatment options. In the 2023 SAMHSA national survey, ~ 2% of individuals aged 12 and older in the United States reported cocaine use within the past year [1]. From the 2019 National Survey on Drug Use and Health, approximately 2.2 million people reported regularly using cocaine of which 1.5 million met the Diagnostic and Statistical Manual for Mental Disorders (DSM-5) criteria for CUD [2, 3]. Among individuals who reported cocaine use, approximately 15% are estimated to progress to CUD within 10 years- the rate of progression higher than that of alcohol (12–13%) and nearly double the rate of cannabis (8%) [2, 4]. Over the past several decades, research has improved our understanding of risk factors contributing to, and brain changes resulting from cocaine use; translation of this knowledge into effective treatment strategies has been less successful. Attempts at direct modulation of the dopamine (DA) system, the primary neurotransmitter associated with reward and mesocorticolimbic function, have not received FDA-approval as treatments for CUD due to lack of efficacy, abuse potential and/or dose-limiting adverse effects resulting in efforts to indirectly modulate DA function [5, 6, 7].

One alternative approach for the treatment of CUD may involve selective modulation of specific muscarinic acetylcholine receptor subtypes. Developments in medicinal chemistry in the past few decades have generated subtype selective ligands targeting the allosteric binding domain of various muscarinic receptor subtypes providing unprecedented ligand-receptor specificity and selectivity with broad-reaching therapeutic applications [8, 9]. There are five known G protein-coupled muscarinic acetylcholine receptor (mAChR) subtypes, termed M_1_-M_5_. Behavioral and neurochemical evidence from preclinical studies support the pharmacotherapeutic potential for CUD via activation/positive allosteric modulation of the M_1_ and/or M_4_ mAChR subtype or inhibition/negative allosteric modulation of the M_5_ mAChR subtype [10, 11]. Specifically, acute administration of M_4_ selective positive allosteric modulators (PAMs) have attenuated cocaine-induced hyperactivity and dopamine efflux in the striatum as well as attenuated cocaine self-administration and discriminative stimulus effects of cocaine in rodents [12, 13]. In humans, limited evidence suggests a polymorphism in the M_4_ mAChR may be associated with CUD [14]. Although early work reported no difference in postmortem M_4_ mAChR mRNA levels from patients with a cocaine (and polysubstance use) history [15], rodent studies confirmed that non-contingent cocaine exposure decreased non-selective muscarinic receptor binding [16, 17, 18]. However, it is unclear how cocaine exposure directly affects M_4_ mAChR receptor expression or availability in a research setting controlling for environmental and social variables.

Non-invasive imaging using positron emission tomography (PET) facilitates quantification of receptor distribution, expression, and activity in vivo under normal and pathological conditions. Recently, [^11^C]MK-6884 was validated as a radiotracer that binds with high affinity (~ 12–17 nM) to an allosteric site on the M_4_ mAChR in vitro and in vivo in rodent, monkey, and human brain tissues, with regional distribution in the brain consistent with M_4_ receptor localization [19, 20, 21]. M_4_ mAChRs are highly expressed in the central nervous system, in the cortex, hippocampus, and striatum [22]. Moreover, human imaging studies demonstrated lower [^11^C]MK-6884 binding potential in the striatum of patients with Alzheimer’s disease compared to healthy-aged humans [21]. The present study directly tested the hypothesis that cocaine self-administration is associated with lower M_4_ mAChR availability using [^11^C]MK-6884 and PET imaging. Rats self-administered cocaine under short (2-h) and longer (4-h) access conditions to ensure a broad range of cocaine intake. Following self-administration, rats were anesthetized and underwent PET imaging with [^11^C]MK-6884 to determine the effects of cocaine exposure on [^11^C]MK-6884 standard uptake values with cerebellum as reference (SUVrs). These PET imaging findings were corroborated with ex vivo autoradiography and post-PET biodistributional analysis.

## Materials and methods

### Subjects

18 male Sprague-Dawley rats (250–300 g; Cohort 1 [*n* = 8]: Envigo, Indianapolis, IN; Cohort 2 [*n* = 10]: Charles River Laboratories, Wilmington, MA) were pair housed in opaque cages (18 in X 10 in X 8 in). All rats had *ad libitum* access to standard rat chow and water, were maintained on a 12-h light/12-h dark cycle, and housed in a temperature and humidity-controlled colony room. All animal care procedures were approved by the Wake Forest University Animal Care and Use Committee (#A23-069; approval date: 07/03/2023). Behavioral studies were initiated during the first half of the 12-h dark phase.

### Cocaine self-administration

Self-administration (SA) procedures have been described previously [23]. Briefly, rats (260–300 g) were implanted with a chronic indwelling jugular vein catheter that was connected to a vascular access button (Instech Labs, Plymouth PA). Following recovery, rats with no prior behavioral history began training to self-administer cocaine (*n* = 4:7; Cohort 1:2) or 45-mg sucrose pellets (Bio-Serve, USA; *n* = 4:3; Cohort 1:2) under a fixed ratio-1 (FR 1) schedule of reinforcement. Cocaine HCl was generously supplied by the NIDA Drug Supply Program. Rats were placed in operant chambers (Med Associates, Fairfax, VT) for cocaine-SA, and were connected to an external infusion pump for drug delivery. Throughout the first 3 sessions, the ratio was increased to a FR 3. For cocaine-SA, rats self-administered 0.5 mg/kg/infusion cocaine for 5 days followed by 0.75 mg/kg/infusion for 10 days. To ensure a large distribution of cocaine intake across the same number of sessions, sessions lasted a maximum length of 2 h or 60 total infusions (Cohort 1) or a maximum session duration of 4 h or a total of 100 cocaine infusions (Cohort 2). All sucrose self-administration sessions (cohorts 1 and 2) had a maximum duration of 2 h or delivery of 50 sucrose pellets. For both cocaine and sucrose pellet SA, reinforcer delivery was paired with a 10-second light cue presentation and retraction of the corresponding lever (10-second timeout).

### [^11^C]MK-6884 synthesis

**Scheme 1:**
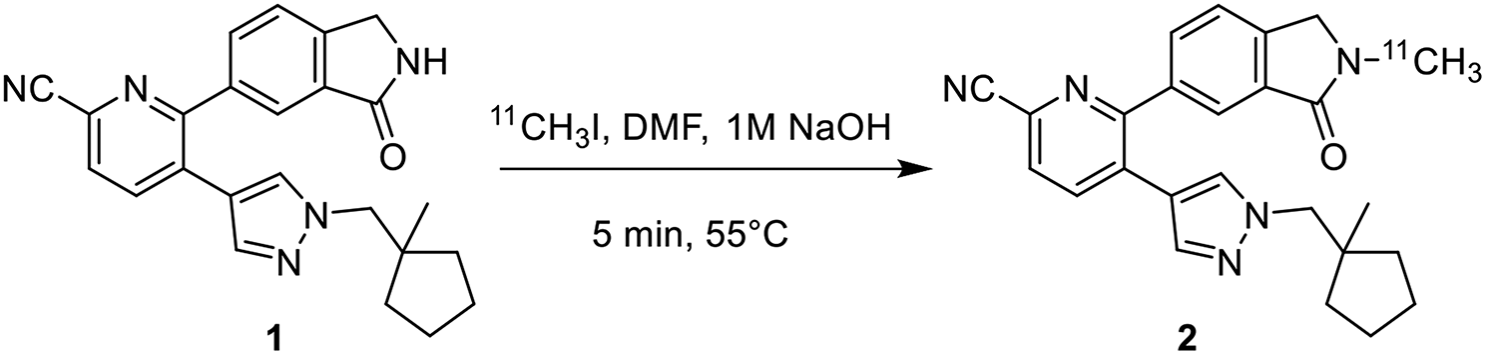
Radiosynthesis of [^11^C]MK-6884

[^11^C]MeI was bubbled to the reaction vial in the GE FXC radiochemistry [24] module containing precursor 1 (1 mg) in anhydrous DMF (0.5 mL) and 1 M NaOH aqueous solution (10 µL) for ∼5 min at room temperature (scheme 1). After the complete transfer of radioactivity, the closed reaction vial was then heated to 55 °C for 5 min. The reaction mixture was quenched with HPLC mobile phase (0.5 mL) and injected onto a reverse-phase semipreparative C18 Phenomenex ODS (250 mm × 10 mm, 10 µ) HPLC column to purify the product [^11^C]MK-6884 [25]. The isocratic HPLC mobile phase solution consisted of 60% acetonitrile, 40% 0.1 M aqueous ammonium formate buffer solution (pH 7.5), with UV λ @ 254 nm and a flow rate of 7 mL/min. The product [^11^C]MK-6884, with the retention time of 10.0–12.0 min was collected and diluted with 25 mL of deionized water and passed through a C18 SepPak cartridge (WAT036800,Waters, Milford, MA) to trap the radioactive product. [^11^C]MK-6884 was then directly eluted from the cartridge with absolute ethanol (1.5 mL) and formulated with saline (10% ethanol in saline) into a sterile vial through a sterile 0.22 μm pyrogen-free filter for further animal studies and quality control analysis. [^11^C]MK-6884 purity was assessed using an analytical Phenomenex C18 HPLC column (250 mm × 4.6 mm, 5 µ) and with UV λ @ 254 nm. The mobile phase (1.0 mL/min) consisted of 60% acetonitrile and 40% 0.1 M aqueous ammonium formate pH 7.0–7.5 solution. The final product was validated by performing a co-injection with the non-radioactive standard MK-6884.

### PET/CT imaging

PET/CT imaging was conducted on all rats (*n* = 11 cocaine and *n* = 7 control) within 24–48 h of the final self-administration session using a TriFoil microPET/CT scanner [21, 26, 27]. Rats were anesthetized with isoflurane (2–3% induction/1–2% maintenance) for the duration of scans. Anesthetized rats underwent immediate dynamic 0–30 min brain PET scans after a tail vein injection of [^11^C]MK-6884 (16.87 ± 2.7 MBq). PET images were reconstructed using the Triumph Scanner software (Trifoil Imaging) with 30 iterations of the Ordered Subsets Expectation Maximization 3D (OSEM-3D) algorithm. Quantitative image analysis was conducted with PMOD software (PMOD technologies, v-4.3 Switzerland). Volumes of interest (VOIs) were drawn for the whole brain, cerebellum, bilateral striatum, bilateral hippocampus, and frontal cortical regions. These VOIs were drawn as contours in high-resolution CT images overlaid with a standard rat brain template [28]. The uptake values of [¹¹C]MK-6884 within each delineated VOI were extracted to calculate standardized uptake values (SUVs) [25]. For further analysis, SUVs were normalized to the cerebellar SUV to generate standardized uptake value ratios (SUVrs). The SUVrs were calculated for the whole brain, bilateral striatum, bilateral hippocampus, and frontal cortex to assess regional tracer uptake differences. SUVrs for the left and right striatum and hippocampus were then averaged.

### Autoradiography

Ex vivo autoradiography studies were performed on frozen brain tissues from a subset of rats (*n* = 3 cocaine-SA, *n* = 3 control group) 24 h post-biodistribution studies to primarily corroborate in vivo PET imaging results following the published protocols [25]. Briefly, coronal Sect. (18 μm thickness) from the frozen brain tissues (48 h-post last cocaine SA session) were mounted on glass slides (Super Frost Plus slides), air-dried for 30 min, and incubated in PBS (pH 7.4) for 10 min. For blocking studies, one set of slides was pre-incubated with 200 µM MK-6884. [^11^C]MK-6884 in PBS (∼0.37 MBq/well) was added to each slide and incubated for 30 min. The slides were then washed with PBS (3X) and water (1X) at 4 °C, and quickly air-dried. Slides containing the radioactive brain tissues were exposed to radioluminographic imaging plates from GE Healthcare with a 12-hour exposure at − 20 °C and scanned with a GE Amersham Typhoon scanner at 25 μm pixel resolution. Autoradiographs were analyzed using ImageQuant TL 8.2 and uptake was calculated as phosphor-stimulated luminescence (PSL/mm^2^).

### Biodistribution

Post-PET tissue biodistribution studies were performed with [^11^C]MK-6884 in the same rats after PET/CT acquisition (*n* = 11 cocaine-SA, *n* = 7 control group). Following the imaging scans, rats were euthanized via cervical dislocation using a rodent guillotine, in accordance with institutional guidelines. Tissues were immediately collected and gamma-counted to obtain biodistribution values. Radioactive uptake in the blood, brain, heart, lungs, liver, spleen, kidney, pancreas, muscle, and bone was calculated as percentages of injected dose per gram of tissue (%ID/g tissue) and with a standard dilution of the injected dose [27].

### Statistical analysis

GraphPad Prism (version 10; GraphPad Software, La Jolla, CA, USA) was used for all statistical analyses. The image analysis team was blinded to all rodent information. For in vivo PET images of the whole brain and autoradiography studies, comparisons were performed using unpaired two-tailed t-tests. For regional analysis of in vivo PET images and biodistribution studies, analyses were performed with two-way ANOVAs using treatment groups and regions as independent variables followed by Tukey’s multiple comparisons test. Correlations between SUVrs and cocaine intake were assessed using separate Pearson’s correlation coefficients (r).

## Results

### Cocaine self-administration

The cumulative cocaine intake across the 15-day self-administration period ranged between 324 and 776 mg/kg (Cohort 1: 324–549 mg/kg; Cohort 2: 375–776 mg/kg). Weights on the last self-administration session were not different between cocaine and sucrose self-administration groups, between cohorts, or between self-administration groups within each cohort (all *p* > 0.05, two-tailed t-tests).

### Radiosynthesis

As shown in scheme 1 the [^11^C]MK-6884 was produced with a radiochemical yield of ~ 15 ± 5%, radiochemical purity of > 95%, and molar activity of ~ 87.71 ± 0.1 GBq/ µmol, decay corrected to the end of synthesis (*n* > 25 runs). [^11^C]MK-6884 demonstrated a retention time of 6.3 min on a QC-HPLC system and authentication of the final product with co-injection of the nonradioactive standard, MK-6884 showed similar retention times.

### PET/CT imaging

**Fig. 1:**
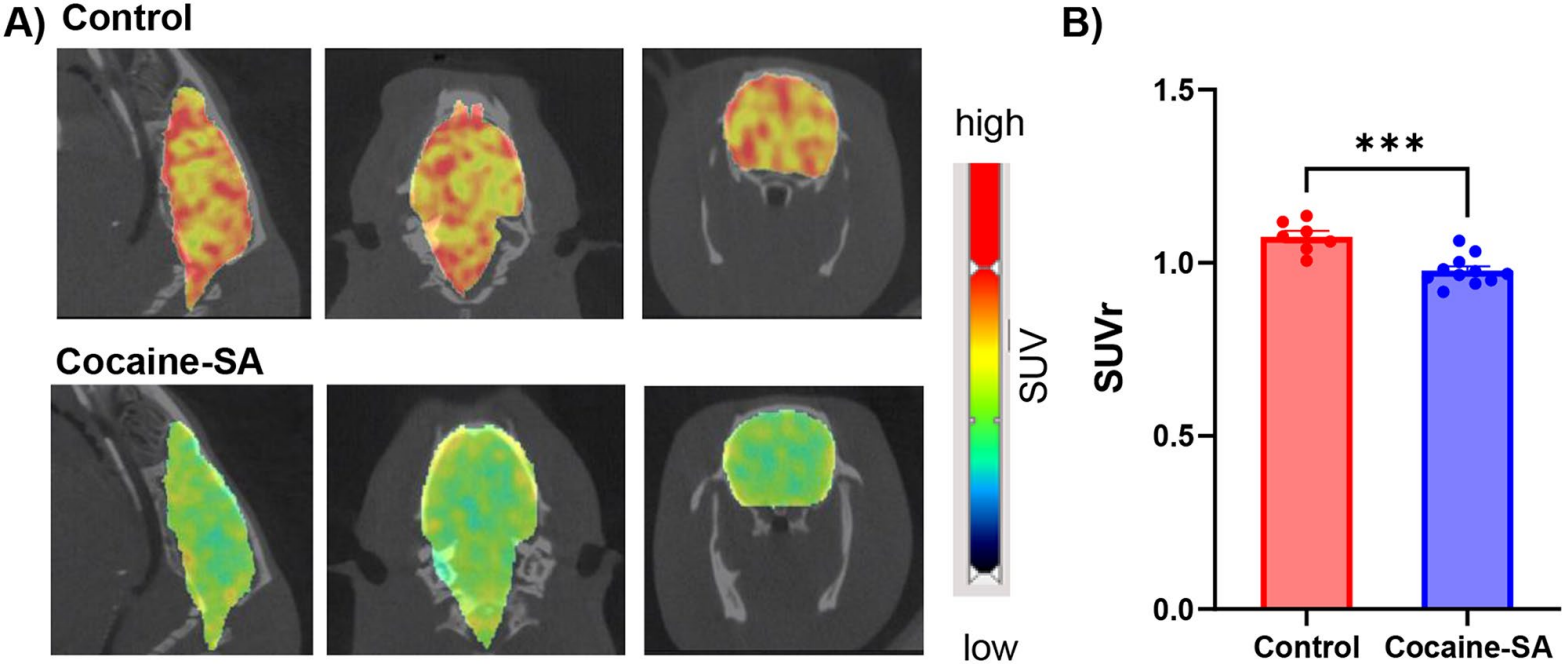
[^11^C]MK-6884 PET imaging in control and cocaine-SA rats. (**A**) Whole-brain sagittal, axial, and coronal views of microPET images overlaid on CT images from a representative control and cocaine self-administration (SA) rat following intravenous injection of [^11^C]MK-6884 (16.87 ± 2.7 MBq). (**B**) Mean (± SEM) whole brain standard uptake values with cerebellum as reference region (SUVrs) of [^11^C]MK-6884 in the control group (*n* = 7) and cocaine self-administration group (Cocaine-SA; *n* = 11). Circles represent individual rats. Statistical analysis was performed using unpaired, two-tailed t-test; *** *p* < 0.001

**Fig. 2:**
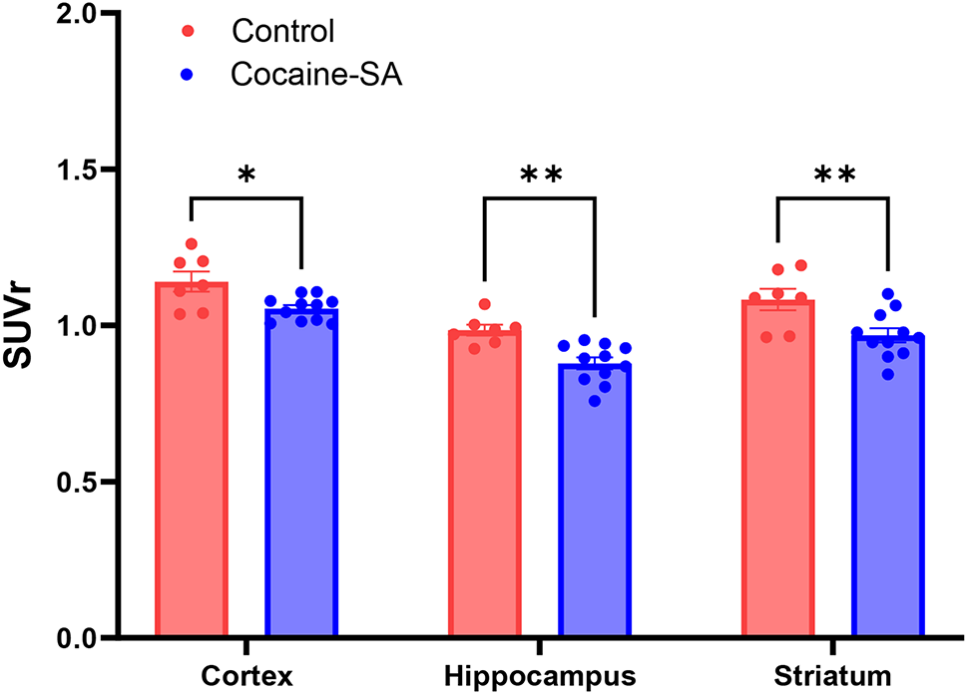
Regional uptake of in vivo [^11^C]MK-6884 PET imaging. Mean (± SEM) standard uptake values with cerebellum as reference region (SUVrs) for cortex, hippocampus, and striatum in the control (*n* = 7) and cocaine self-administration group (Control-SA; *n* = 11). Circles represent individual rats. Statistical analysis was performed using two-way ANOVA followed by Tukey’s multiple comparisons test; *p < 0.05, **p < 0.01

**Fig. 3:**
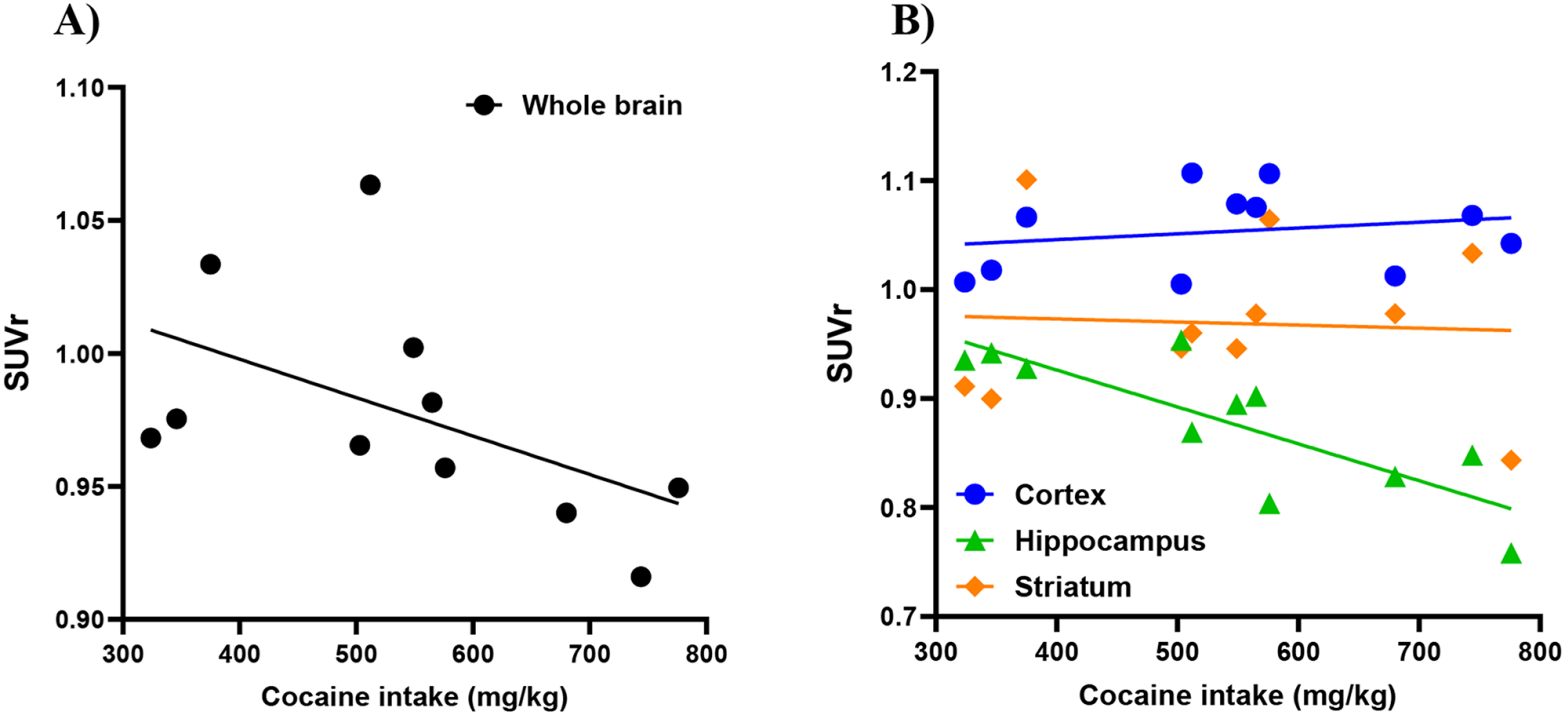
Correlation between cocaine intake and in vivo [^11^C]MK-6884 PET imaging. Correlation between cumulative cocaine intake (mg/kg) and standard uptake values from in vivo [^11^C]MK-6884 PET imaging using cerebellum as reference region (SUVr) in (**A**) whole brain and (**B**) in cortex, hippocampus, and striatum. Individual data points (*n* = 11) were fitted by linear regression. Pearson’s correlation coefficients for whole brain (*r* = -0.522, *p* = 0.099), cortex (*r* = 0.211, *p* = 0.532), hippocampus (*r* = -0.819, *p* < 0.01), and striatum (*r* = -0.057, *p* = 0.866)

The injection of [^11^C]MK-6884 (16.87 ± 2.7 MBq) into both control and cocaine-SA rats showed rapid penetration of the tracer across the blood-brain barrier. Time-activity curves for the control group, presented in Supplementary Figure S1, confirmed robust tracer kinetics. Whole brain SUVrs analysis showed a significant difference (t_16_ = 4.71,*** *p* < 0.001) between cocaine-SA (*n* = 11) compared to control rats (*n* = 7) (Fig. 1A &B). Two-way ANOVA revealed a significant effect of treatment (F_(1,48)_ = 29.72; *p* < 0.001) and region (F_(2,48)_ = 25.94; *p* < 0.001) but not an interaction (F_(2,48)_ = 0.1825; *p* > 0.05). Tukey’s post hoc regional analysis of [^11^C]MK-6884 revealed lower SUVrs in the striatum, (*p < 0.01), cortex (*p < 0.05), and hippocampus (*p < 0.01) of cocaine-SA rats compared to control rats (Fig. 2). Within cocaine-SA rats, there was a trend toward lower [^11^C]MK-6884 SUVrs associated with greater cocaine intake across the whole brain (Pearson *r* = -0.522; *p* = 0.099) (Fig. 3A), striatum (*r* = -0.057, *p* = 0.866), and cortex (*r* = 0.211, *p* = 0.532), with a significant negative correlation between cocaine intake and [^11^C]MK-6884 SUVrs in the hippocampus (*r* = -0.819, **p < 0.01) (Fig. 3B).

### Ex vivo autoradiography studies

**Fig. 4:**
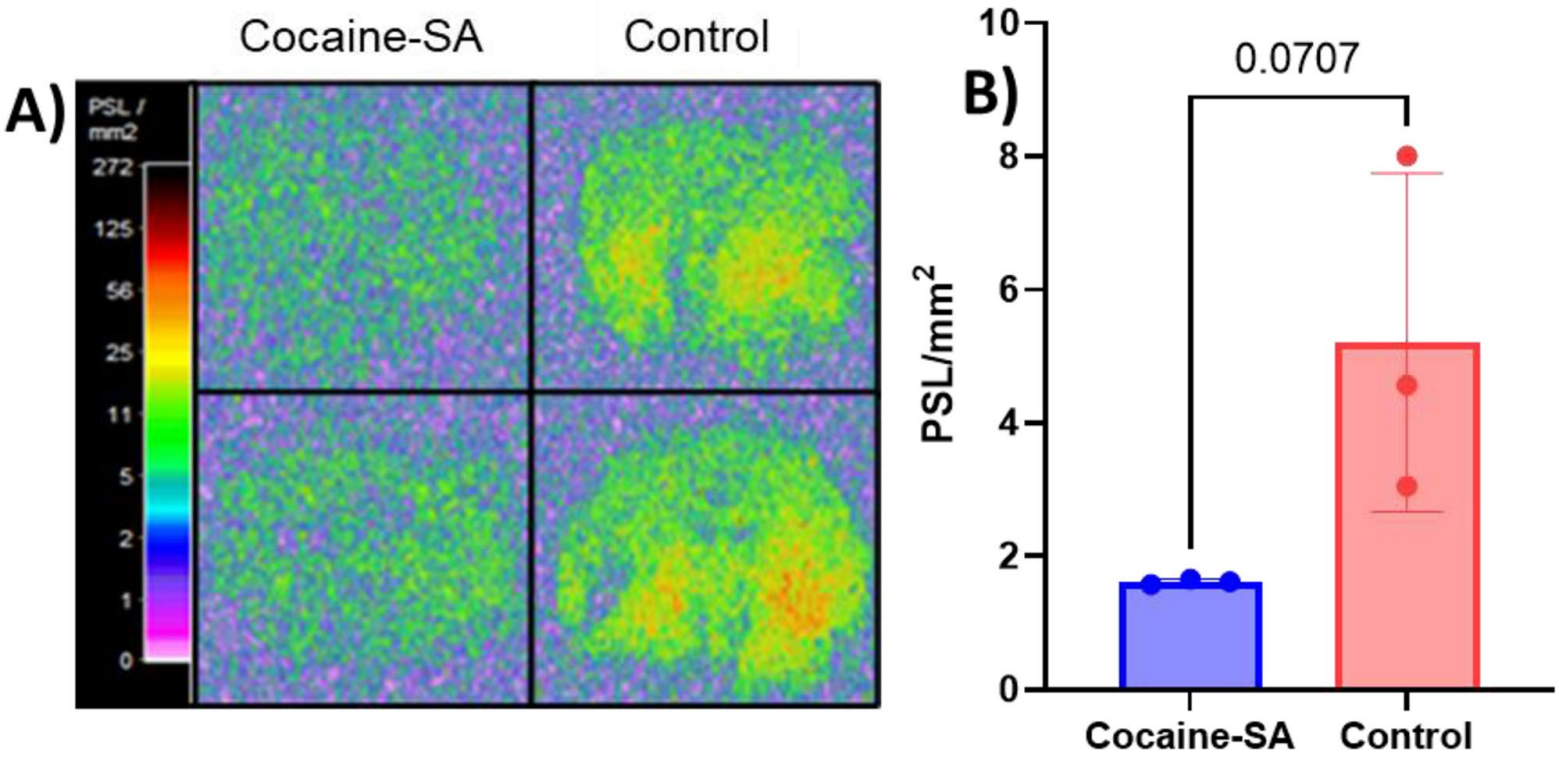
Ex vivo autoradiography of [^11^C]MK-6884. (**A**) Visualization and (**B**) quantification (mean ± SD) of [^11^C]MK-6884 uptake in identical coronal brain sections of the cocaine self-administration group (cocaine-SA; *n* = 3) and the control group (*n* = 3) was calculated as phosphor-stimulated luminescence values (PSL/mm^2^). Statistical analysis was performed using unpaired, two-tailed t-test; *p* = 0.0707

Autoradiography studies on coronal sections of brain tissue from a subset of cocaine-SA and control rats with [^11^C]MK-6884 radiotracer corroborated the findings of PET imaging. The signal intensity of autoradiograms of the cocaine-SA group (*n* = 3) was lower (*p* = 0.07) compared to the control group (*n* = 3) as shown in Fig. 4A & B. Although not statistically significant (likely due to small sample size) these data suggest lower M_4_ mAChR density and availability in the cocaine-SA group. Additionally, the blocking study demonstrated a 2.3-fold decrease in signal intensity relative to controls (p = **0.0034).

### Biodistribution studies

Biodistribution studies offer a comprehensive and additional evaluation of [^11^C]MK-6884’s uptake, tissue localization, and clearance using ex vivo analyses for assessment in both cocaine-SA and control rats. [^11^C]MK-6884 radiotracer exhibited a nominal uptake into heart, lungs, spleen, muscle, blood, and bone, and increased uptake in brain, liver, and pancreas in both cocaine-SA and control rats (Table 1). Two-way ANOVA revealed a significant effect of brain region (F _(9,160)_ = 192.7; *p* < 0.001) but not treatment (F_(1,160)_ = 3.05; *p* = 0.08) nor an interaction (F_(9,160)_ = 1.902; *p* = 0.055). Importantly, post-hoc analysis demonstrated significantly lower [^11^C]MK-6884 uptake in the whole brain of the cocaine-SA group versus the control group (*p < 0.05) but not peripheral regions (all *p* > 0.05). Thus, brain distribution data of [^11^C]MK-6884 ex vivo (Table 1) corroborate well with the in vivo PET imaging data (Fig. 1B), further validating our significant findings.

**Table 1:** Biodistribution of [^11^C]MK-6884 in cocaine-SA and control rats^a^

organ	cocaine-SA rats	control rats
blood	0.40 ± 0.11	0.56 ± 0.20
brain*	0.89 ± 0.13	1.77 ± 0.68
heart	0.50 ± 0.14	0.56 ± 0.17
lungs	0.55 ± 0.14	0.67 ± 0.18
liver	5.95 ± 0.13	6.55 ± 0.18
spleen	0.59 ± 0.17	0.58 ± 0.20
kidney	5.17 ± 0.51	4.48 ± 1.02
pancreas	0.93 ± 0.26	1.38 ± 0.31
muscle	0.27 ± 0.18	0.30 ± 0.31
bone	0.42 ± 0.13	0.50 ± 0.19

^a^Values were reported as percentages of the injected dose per gram of tissue (%ID/g) ± SD, in brain uptake between cocaine self-administration (cocaine-SA; *n* = 11) and control (*n* = 7) rats. Statistical analysis was performed using a two-way ANOVA followed by Tukey’s multiple comparisons test; *p < 0.05 between groups

## Discussion

Despite recent evidence that modulation of the cholinergic system may have therapeutic potential for the treatment of cocaine and other substance use disorders, scant research has directly examined cholinergic integrity, specifically concerning mAChR function or receptor distribution, in the context of substance use disorders [29]. Although recent findings report reduced M_4_ mAChR expression (mRNA and protein) in rats following long-term ethanol exposure and abstinence, and in the striatum of human post-mortem tissue from humans diagnosed with alcohol use disorder [30, 31] to our knowledge similar studies have not been conducted in the context of cocaine use disorder (CUD). The present study established an association between cocaine intake and M_4_ mAChR availability and distribution. This work extends prior ex vivo research describing global reductions in mAChR distribution [16, 17, 18] and confirms specific alterations of the M_4_ mAChR subtype. These data align with preclinical studies demonstrating that enhancing M_4_ mAChR function via positive allosteric modulators decreases cocaine-induced alterations in neurochemistry and behavior, notably cocaine self-administration [14, 15].

[^11^C]MK-6884 is a novel PET ligand that binds with high affinity to an M_4_ mAChR allosteric binding site. Prior studies have confirmed high binding potential in the M_4_ mAChR-rich regions including the striatum, cortex, hippocampus, and thalamus across mouse, monkey and human brain tissue that can be dose-dependently blocked by administration of an M_4_ PAM [32]. Interestingly, prior studies have shown that the addition of either an acetylcholine esterase inhibitor or a cholinergic agonist binding to an orthosteric binding site was shown to increase [^11^C]MK-6884 binding potential [20, 21] reiterating the unique receptor binding profile of allosteric modulators vs. classic orthosteric ligands. Thus, lower whole brain M_4_ mAChR SUVrs following cocaine SA could be affected by altered endogenous cholinergic tone or M_4_ mAChR distribution.

Importantly, autoradiography studies suggest lower M_4_ mAChR density in a subset of rats with a cocaine SA history. A direct comparison between the autoradiography and PET study results further emphasizes these findings. The PET imaging results showed a significant reduction in [^11^C]MK-6884 SUVrs in key brain regions, including the cortex, striatum, and hippocampus, in the cocaine-SA group, corroborated by autoradiography data demonstrating decreased signal intensity. Due to the limited resolution of autoradiography films, however, regions of interest (ROIs) could not be defined identically to those in PET imaging. Despite this limitation and a small sample size, the observed reduction in autoradiography signal intensity in the cocaine-SA group indicates lower M_4_ mAChR availability consistent with the PET findings. Additionally, biodistribution data indicated a significant decrease in [^11^C]MK-6884 uptake in the whole brain of cocaine-SA rats, aligning with the region-specific decreases observed in PET imaging. Together, the results from PET, autoradiography, and biodistribution support a reduction in M_4_ mAChR density and availability following cocaine exposure, underscoring altered receptor expression within the cholinergic system in the context of CUD.

The present study provides a clear direction for future research. Present studies were limited to male rats that only self-administered cocaine for 15 days. Given known sex differences in drug-related behaviors (e.g. sensitivity to reinforcing and discriminative stimulus effects, pharmacokinetics) and effects of the menstrual cycle phase on receptor distribution [33, 34, 35, 36, 37], future studies with female rats are needed. Additional longitudinal, within-subject studies are needed to confirm a causal role of cocaine SA on M_4_ mAChR availability, extending beyond 15 days of self-administration, as well as recovery following extended periods of abstinence (and perhaps to examine basal levels as a biomarker of vulnerability to CUD). Regardless, PET imaging with [^11^C]MK-6884 provides strong translational potential for parsing mAChR subtype-selective alterations associated with CUD that can broadly be extended to other substance/alcohol use disorders and CNS disorders in general.

The cholinergic system has been a longstanding target for investigating novel treatments for Alzheimer’s disease and schizophrenia [8], and has only more recently been investigated in disorders of motor function, psychostimulant, opioid, and alcohol use disorders [11, 38, 39, 40]. Initial studies implicated a role in M_4_ modulation of striatal DA function and reinforcement-related behaviors. For example, mice in which the M_4_ mAChR was genetically deleted (M_4_ KO) reported higher cocaine-induced locomotion and extracellular striatal dopamine release as well as greater cocaine intake and higher breakpoints in cocaine self-administration studies compared to wild-type mice [41]. M_4_ PAMs have been shown to attenuate cocaine self-administration as well as the discriminative stimulus effects of cocaine. However, the present study is the first to report lower striatal M_4_ distribution following cocaine self-administration.

Cholinergic modulation has also been shown to impact arousal states, executive function, and sleep [42, 43, 44], each of which is negatively impacted by cocaine exposure [45, 46] and modulated by M_4_ mAChR function [43, 47, 48]. M_4_ PAMs have been shown to impact hippocampal synaptic transmission and improve hippocampal- and cortical-dependent learning and memory in rodents [47, 48, 49]. Lower M_4_ mAChR distribution in the hippocampus and cortex (and globally) may suggest an etiological role for M_4_ mAChR function contributing to other symptoms associated with CUD. Although speculative, future studies are needed to test the hypothesis that M_4_ PAMs may have broad therapeutic potential in treating multiple symptoms, including DSM-5 recognized stimulant-induced disorders (e.g. cognitive and sleep impairments, anxiety, anhedonia). Evaluating alterations in mAChR distribution and/or function, specifically the M_4_ mAChR, is critical given the recent development of subtype-selective allosteric modulators [50] and FDA-approval of KarXT, a first-in-class treatment for schizophrenia that demonstrates antipsychotic efficacy by preferentially activating M_1_/M_4_ mAChRs [51]. In fact, xanomeline, the CNS penetrant compound in KarXT has attenuated cocaine and ethanol-related behaviors in preclinical models and thus, may hold promise as a potential treatment for cocaine and alcohol use disorders [30, 31, 39, 52, 53]. Disentangling the mAChR subtype-specific neurobiological alterations following drug exposure and how that impacts pharmacological efficacy is critical for understanding the treatment potential of mAChR ligands for cocaine and other substance/alcohol use disorders.

## Conclusion

Present studies with the [^11^C]MK-6884 radiotracer indicate that cocaine self-administration is associated with lower M_4_ mAChR distribution and reiterate the pharmacotherapeutic potential of M_4_ mAChR agonism/positive allosteric modulation. Building on prior research through which enhancement of M_4_ mAChR function may have pharmacotherapeutic utility for CUD, present studies suggest restoring cocaine-related deficits in endogenous cholinergic function may also be relevant. Moreover, present findings suggest additional putative therapeutic benefits for CUD that may accompany enhancing M_4_ mAChR function, which should be considered in future research.

## Electronic supplementary material

Below is the link to the electronic supplementary material.

Supplementary Material 1

## Data Availability

Data is provided within the manuscript. Additional access to raw data is available upon request via the corresponding authors.

## Abbreviations

CT: Computed Tomography
CUD: Cocaine Use Disorder
DA: Dopamine
DSM-5: Diagnostic and Statistical Manual for Mental Disorders-Version 5
FDA: United States Food and Drug Administration
FR: Fixed Ratio
H: Hour
HCl: Hydrochloride
HPLC: High Performance Liquid Chromatography
KO: Knockout
M: Molar
mAChR: muscarinic Acetylcholine Receptor
mg/kg: Milligram/Kilogram
min: Minute
mL: Milliliter
mRNA: Messenger Ribonucleic Acid
NIDA: National Institute on Drug Abuse
OSEM-3D: Ordered Subsets Expectation Maximization 3D
PAM: Positive Allosteric Modulator
PBS: Phosphate Buffered Saline
PET: Positron Emission Tomography
ROI: Region Of Interest
SA: Self-Administration
SAMHSA: Substance Abuse and Mental Health Services Administration
SUV: Standard Uptake Value
SUVr: Standard Uptake Value with cerebellum as reference region
uL: microliter
UV: Ultraviolet
VOI: Volume Of Interest
%ID/g: Percent Injected Dose/gram of tissue
