# Supplementary material for: [11C]MK-6884 PET imaging reveals lower M4 muscarinic acetylcholine receptor availability following cocaine self-administration in male rats
Source: Pharmacol Rep. 2025 Jan 14;77(2):532–41. doi: 10.1007/s43440-025-00695-9 (PMC11911261; doi:10.1007/s43440-025-00695-9)
Supplement: Supplementary file 1 — Supplementary Material 1 [file 43440_2025_695_MOESM1_ESM.docx]

**Supplementary Information**

**[^11^C]MK-6884 PET imaging reveals lower M_4_ muscarinic acetylcholine receptor availability following cocaine self-administration in male rats**

Krishna K Gollapelli^1+^, Ivan Krizan^1+^, Bhuvanachandra Bhoopal,^1+^ Naresh Damuka^1^, Carson Moriarty^2^, Mack Miller^1^, Kiran K. Solingapuram Sai^1,2^^, Robert W. Gould^2^^

^1^Department of Radiology, Wake Forest University School of Medicine, Winston-Salem, NC, USA

^2^Department of Translational Neuroscience, Wake Forest University School of Medicine, Winston-Salem, NC, USA

^+^Authors Contributed Equally

^^^Co-corresponding Authors:

Robert Gould, PhD

Associate Professor

Department of Translational Neuroscience, Center for Addiction Research

Wake Forest University School of Medicine, Winston-Salem, NC, USA

115 South Chestnut St.

Winston-Salem, NC 27101

Email: rgould@wakehealth.edu

Kiran K Solingapuram Sai, PhD

Associate Professor

Department of Radiology and Translational Neuroscience, Center for Addiction Research

Wake Forest University School of Medicine, Winston-Salem, NC, USA

Email: ksolinga@wakehealth.edu

Running Title: Cocaine-associated decreases in M_4_ mAChR distribution

**Figure S1.** Average time activity curve of [^11^C]MK-6884 in the control group (Con; n=7) rats.
